# A joint alignment and reconstruction algorithm for electron tomography to visualize in-depth cell-to-cell interactions

**DOI:** 10.1007/s00418-022-02095-z

**Published:** 2022-03-23

**Authors:** Lea Bogensperger, Erich Kobler, Dominique Pernitsch, Petra Kotzbeck, Thomas R. Pieber, Thomas Pock, Dagmar Kolb

**Affiliations:** 1 Institute of Computer Graphics and Vision, Graz University of Technology, Graz, Austria; 2 Institute of Computer Graphics, University of Linz, Linz, Austria; 3 Core Facility Ultrastructure Analysis, Neue Stiftingtalstraße 6/II, 8010 Graz, Austria; 4 Gottfried Schatz Research Center for Cell Signaling, Metabolism and Aging, Division of Cell Biology, Histology and Embryology, Medical University of Graz, Neue Stiftingtalstraße 6/II, 8010 Graz, Austria; 5 COREMED, Cooperative Centre for Regenerative Medicine, Joanneum Research Forschungsgesellschaft mbH, Graz, Austria; 6 Division of Endocrinology and Diabetology, Medical University of Graz, Graz, Austria; 7 The Research Unit for Tissue Regeneration, Repair and Reconstruction, c/o Division of Plastic, Aesthetic and Reconstructive Surgery, Department of Surgery, Medical University of Graz, Graz, Austria; 8 The Center for Biomarker Research in Medicine GmbH, Graz, Austria

**Keywords:** Electron microscopy, Electron tomography, Joint alignment and reconstruction algorithm, Fiducial-less alignment, Primal-dual iterative reconstruction, Inverse problem

## Abstract

Electron tomography allows one to obtain 3D reconstructions visualizing a tissue’s ultrastructure from a series of 2D projection images. An inherent problem with this imaging technique is that its projection images contain unwanted shifts, which must be corrected for to achieve reliable reconstructions. Commonly, the projection images are aligned with each other by means of fiducial markers prior to the reconstruction procedure. In this work, we propose a joint alignment and reconstruction algorithm that iteratively solves for both the unknown reconstruction and the unintentional shift and does not require any fiducial markers. We evaluate the approach first on synthetic phantom data where the focus is not only on the reconstruction quality but more importantly on the shift correction. Subsequently, we apply the algorithm to healthy C57BL/6J mice and then compare it with non-obese diabetic (NOD) mice, with the aim of visualizing the attack of immune cells on pancreatic beta cells within type 1 diabetic mice at a more profound level through 3D analysis. We empirically demonstrate that the proposed algorithm is able to compute the shift with a remaining error at only the sub-pixel level and yields high-quality reconstructions for the limited-angle inverse problem. By decreasing labour and material costs, the algorithm facilitates further research directed towards investigating the immune system’s attacks in pancreata of NOD mice for numerous samples at different stages of type 1 diabetes.

**Supplementary Information:**

The online version contains supplementary material available at 10.1007/s00418-022-02095-z.

## Introduction

Electron tomography is an imaging technique based on transmission electron microscopy (TEM) that enables the investigation of 3D cellular and molecular structures and interactions. A series of projection images is acquired by tilting a sample between approximately −60 and +60 degrees, which is followed by reconstructing the volume of interest from the tilt series. Tilting the imaged object to angles larger than ±60$^\circ$ is seldom done in practice, because the path that electrons travel through the biological specimen would be much longer owing to increased thickness at higher tilt angles, which obstructs the transmitting electrons. This is also known as the missing wedge effect since a substantial amount of information in the Fourier domain cannot be measured (Frank 2006). Hence, the reconstruction of a tilt series is a limited angle problem, similar to limited angle in computed tomography. A major issue of the measurement process is the sensitivity to instabilities in the microscope’s specimen stage during tilting but also to external environmental influences such as mechanical vibrations. These interfering factors lead to inaccuracies such as distinct translations among each projection image due to the tilting of the goniometer or shearing caused by the interaction of incident electrons with biological matter.

To enable an accurate reconstruction process, the projection images must be aligned with each other. The accuracy of the alignment process highly influences the quality and resolution of the reconstruction. The gold standard used for alignment of biological samples is to label each sample with gold particles prior to the acquisition process. The fiducial gold markers are clearly distinguishable in their intensity in the projection images and thus allow for a global alignment. While this has already been proposed in the 1980s by Berriman et al. (1984), it prevails as a very commonly used practice (Mastronarde 2006; Han et al. 2017) and is implemented in the IMOD package (Kremer et al. 1996), an open-source programme for 3D reconstructions. Labelling the tissue with gold particles must be done manually and might induce streaking artefacts in the reconstruction, thus potentially covering areas of interest. Furthermore, it is a time-consuming process that relies on costly material. In contrast, there are also fiducial-less alternative alignment strategies. Starting as early as 1989, Dengler (1989) addressed the problem using a coarse-to-fine bootstrap control strategy and thus paved the way for iterative reconstruction and alignment methods. There are various different iterative approaches that can broadly be divided into alignment based on projection matching and cross-correlation. Yang et al. (2005) propose an alignment technique by projection matching using a quasi-Newton algorithm to iteratively search for optimal translation parameters. On the other hand, cross-correlation is also used to align projection images iteratively paired with alternating reconstruction methods (Frank and McEwen 1992; Owen and Landis 1996; Gürsoy et al. 2017).

The second aspect in recovering the underlying sample apart from a suitable alignment of the projection images is the reconstruction procedure. There are a variety of reconstruction techniques that can generally be classified into analytical and algebraic methods. Filtered back-projection (FBP) is considered to be the standard analytical method for tomographic reconstruction, but the increase in computational power has led to a more frequent use of algebraic methods such as the algebraic reconstruction technique (ART) and the simultaneous iterative reconstruction technique (SIRT) (Gordon et al. 1970; Sluis and Vorst 1990; Sorzano et al. 2017).

In this work, we propose a fiducial-less alignment and reconstruction algorithm (FLARA) for electron tomography that jointly solves for the unknown reconstruction volume and the disruptive shift. The reconstruction is based on an iterative primal-dual algorithm, which enables an effective shift computation. In contrast to existing fiducial-less approaches, the shift is refined by solving a linearized approximation in each iteration. The limited angle reconstruction process is further improved using total variation regularization, a very common choice for regularization in imaging problems that was introduced already in 1992 by Rudin et al. (1992). As a result, no fiducial markers are needed for alignment, thus saving material and labour costs.

The proposed algorithm FLARA is suitable for any limited angle problem accompanied by translatory shifts in the measurement data. We plan to use it in future research uncovering cellular interactions in pancreatic islets in clinical and preclinical models of type 1 diabetes. Obtaining 3D reconstructions therefore provides the possibility of identifying first interactions between beta cells and immune cells, which initiates the autoimmune response leading to full destruction of beta cells, diminished insulin production and onset of type 1 diabetes (Roep et al. 2021). This will give insight into the dynamic immune cell attack on beta cells, including the role and fate of the insulin-secretory granules, and can thereby aid in the design of better approaches tailored to type 1 diabetes treatment. To set up and test the newly designed algorithm, tomograms from healthy pancreatic islets from C57BL/6J mice were used and compared with islets from non-obese diabetic mice (NOD/ShiLtJ), which are widely used in type 1 diabetic research (Mathews et al. 2015). The fact that no fiducial markers are needed while the alignment problem requires only computation of 2D translation vectors facilitates the algorithm’s application for larger amounts of NOD samples to visualize the immune system’s attack at different stages for several islets.

## Materials and methods

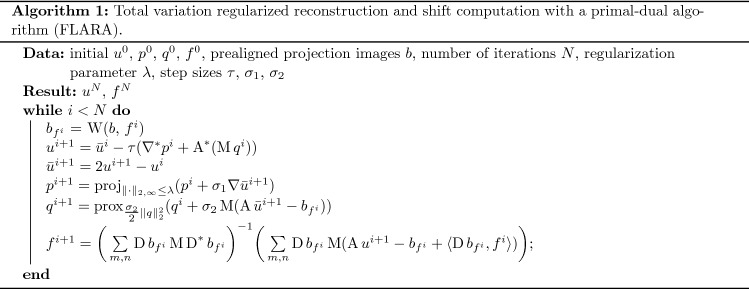



In the following section, the alignment and reconstruction problem and the proposed algorithm FLARA used to solve it are explained. In addition, the dataset and instrumentation are defined.

### Joint alignment and reconstruction

#### Reconstruction problem

Reconstructing inverse problems is the process of recovering the underlying data that led to a given observation. A linear inverse problem is generally formulated as

1 $$\begin{aligned} {{\,\mathrm{\mathrm {A}}\,}}u = b, \end{aligned}$$

where *b* is measured observations, the linear operator ${{\,\mathrm{\mathrm {A}}\,}}$ models a forward operation, and *u* is the ground truth data that have to be recovered. In the available case of electron tomography, the dimensions *z*, *m* and *n* denote the size of the 3D object $u\in {{\,\mathrm{\mathbb {R}}\,}}^{zmn}$ that was imaged, where *z* represents the number of slices of size $m\times n$, and $n_{\alpha }$ represents the number of tilt angles used in the measurement. Thus, the measured observation $b\in {{\,\mathrm{\mathbb {R}}\,}}^{n_{\alpha }mn}$ is the tilt series and the forward operator ${{\,\mathrm{\mathrm {A}}\,}}: {{\,\mathrm{\mathbb {R}}\,}}^{z m n} \rightarrow {{\,\mathrm{\mathbb {R}}\,}}^{n_{\alpha }m n}$ models the projection operation arising from the discretized Radon transform. The corresponding adjoint operator is denoted by ${{\,\mathrm{\mathrm {A}}\,}}^*$. There are two issues that impede a direct reconstruction of Equation (1). Firstly, the unintended shift in the projection images significantly deteriorates the reconstruction and has to be corrected before or during the reconstruction process. Moreover, the fact that ${{\,\mathrm{\mathrm {A}}\,}}$ only covers a tilt angle span of up to $\pm 60^\circ$ leads to the aforementioned limited angle problem, which is ill-posed. A substantial amount of information in the Fourier domain is not captured, and reconstruction artefacts will originate from the missing wedge.

This effect can be diminished to some extent by regularizing the ill-posed inverse problem, thus introducing prior knowledge of the sample of interest into the reconstruction problem. Here, we facilitate the well-studied total variation regularizer, which is a simple but effective regularizer that enforces sparsity in the gradients of the reconstruction. The reconstruction of the 3D volume $u \in {{\,\mathrm{\mathbb {R}}\,}}^{zmn}$ and the shift $f \in {{\,\mathrm{\mathbb {R}}\,}}^{2 \times n_{\alpha }}$, which is two-dimensional per projection image, is cast as a joint minimization problem composed of a data fidelity and a regularization term

2 $$\begin{aligned} \min _{\begin{array}{c} u\in {{\,\mathrm{\mathbb {R}}\,}}^{zmn},\\ f \in {{\,\mathrm{\mathbb {R}}\,}}^{2 \times n_{\alpha }} \end{array}} \tfrac{1}{2} \Vert {{\,\mathrm{\mathrm {M}}\,}}( {{\,\mathrm{\mathrm {A}}\,}}u - {{\,\mathrm{\mathrm {W}}\,}}(b,f))\Vert _2^2 + \lambda \Vert \nabla u\Vert _{2,1} , \end{aligned}$$

where $\nabla : {{\,\mathrm{\mathbb {R}}\,}}^{z m n} \rightarrow {{\,\mathrm{\mathbb {R}}\,}}^{3 \times z m n}$ is the 3D finite difference operator. The warping operator ${{\,\mathrm{\mathrm {W}}\,}}: {{\,\mathrm{\mathbb {R}}\,}}^{n_{\alpha }m n}\times {{\,\mathrm{\mathbb {R}}\,}}^{2\times n_\alpha } \rightarrow {{\,\mathrm{\mathbb {R}}\,}}^{n_{\alpha }m n}$ translates the projection images $b\in {{\,\mathrm{\mathbb {R}}\,}}^{n_{\alpha }mn}$ by shifts $f\in {{\,\mathrm{\mathbb {R}}\,}}^{2\times n_\alpha }$. The mask ${{\,\mathrm{\mathrm {M}}\,}}: {{\,\mathrm{\mathbb {R}}\,}}^{n_{\alpha }m n} \rightarrow {{\,\mathrm{\mathbb {R}}\,}}^{n_{\alpha }m n}$ is applied pixel-wise to exclude not reconstructed regions on the boundary of the projection images depending on the tilt angle, thus compensating for the fact that higher tilt angles imply a compression of the imaged structures within the projection images. The parameter $\lambda \in {{\,\mathrm{\mathbb {R}}\,}}^+$ controls the influence of the regularizer with respect to the data fidelity term. The ASTRA toolbox (Aarle et al. 2015) is used to model the forward and backward projections ${{\,\mathrm{\mathrm {A}}\,}}$ and ${{\,\mathrm{\mathrm {A}}\,}}^*$. In this model, the projection images are assumed to have an underlying translation, which is a global translation $f_{\alpha _i}\in {{\,\mathrm{\mathbb {R}}\,}}^2$ per projection image $b_{\alpha _i} \in {{\,\mathrm{\mathbb {R}}\,}}^{mn}$, which shifts the projections in either of the two planar dimensions. This is a reasonable assumption, since by virtue of the orthonormal projection only the projected 3D translation – a 2D translation in the projection plane – can be measured. Furthermore, the sample was exposed to electron radiation before acquiring the tilt series, a process which is often referred to as ‘baking’ (Pinali and Kitmitto 2014). This ensures sufficient stability during the measurement process and prevents any beam damage and subsequent shearing processes.

In a pre-processing step, the projection images are aligned using cross-correlation between two successive projections starting from the 0$^\circ$ projection image. Each projection image is dilated with the cosine of its respective tilt angle (Dierksen et al. 1992) to compensate for compression of the object’s spatial dimensions at higher tilt angles, since the electron beam and detector position remain unchanged whilst the object is tilted. Since the remaining shifts in the projection images are small, it is justifiable to linearize ${{\,\mathrm{\mathrm {W}}\,}}(b,f)$ with a first-order Taylor expansion (Mastronarde 1981) such that

3 $$\begin{aligned} {{\,\mathrm{\mathrm {W}}\,}}(b,f) \approx b_{f^i} + \langle {{\,\mathrm{\mathrm {D}}\,}}b_{f^i}, f-f^i\rangle , \end{aligned}$$

where $b_{f^i}={{\,\mathrm{\mathrm {W}}\,}}(b,f^i)$ are the projection images shifted by an estimate of the shift $f^i \in {{\,\mathrm{\mathbb {R}}\,}}^{2 \times n_{\alpha }}$. The central difference operator ${{\,\mathrm{\mathrm {D}}\,}}: {{\,\mathrm{\mathbb {R}}\,}}^{n_{\alpha } m n} \rightarrow {{\,\mathrm{\mathbb {R}}\,}}^{2 \times n_{\alpha } m n}$ is used to obtain the currently shifted projection images’ spatial gradients ${{\,\mathrm{\mathrm {D}}\,}}b_{f^i}$. It is crucial to use a finite difference operator based on central differences to ensure that the resulting pixel grid positions match the position of the shift vector. Inserting the linearized warping operator from Equation (3) into the joint alignment and reconstruction problem in Equation (2) yields the following minimization problem

4 $$\begin{aligned} \min _{\begin{array}{c} u\in {{\,\mathrm{\mathbb {R}}\,}}^{zmn},\\ f \in {{\,\mathrm{\mathbb {R}}\,}}^{2 \times n_{\alpha }} \end{array}}&\tfrac{1}{2} \Vert {{\,\mathrm{\mathrm {M}}\,}}( {{\,\mathrm{\mathrm {A}}\,}}u - {{\,\mathrm{\mathrm {W}}\,}}(b,f^i) - \langle {{\,\mathrm{\mathrm {D}}\,}}{{\,\mathrm{\mathrm {W}}\,}}(b,f^i), f-f^i\rangle )\Vert _2^2 +\nonumber \\&\lambda \Vert \nabla u\Vert _{2,1}, \end{aligned}$$

which can be solved iteratively for $i \in \{1,...,N\}$ iterations.

Note that the minimization with respect to *f* and *u* can be split into two separate sub-problems defined in the next sections, where one variable is held constant while solving for the other in an alternating manner.

#### Shift sub-problem

The first sub-problem targets the computation of the unknown shift *f* and is obtained by considering only the data fidelity term in Equation (4) since the regularization term does not contain the shift. It reads as

5 $$\begin{aligned} \min _{f \in {{\,\mathrm{\mathbb {R}}\,}}^{2 \times n_{\alpha }}}\big \{g(f) :=\tfrac{1}{2} \Vert {{\,\mathrm{\mathrm {M}}\,}}( {{\,\mathrm{\mathrm {A}}\,}}u - b_{f^i} - \langle {{\,\mathrm{\mathrm {D}}\,}}b_{f^i}, f-f^i\rangle )\Vert _2^2\big \}, \end{aligned}$$

where the previously estimated shift is denoted by $f^i$. Recall that $b_{f^i}={{\,\mathrm{\mathrm {W}}\,}}(b,f^i)$ denotes the translated projection images. The first-order optimality condition $\frac{\partial g}{\partial f} {\mathop {=}\limits ^{!}} 0$ can be used to solve for the optimal shift *f* given the reconstruction *u*. The expression to compute the new shift estimate $f^{i+1}$ then reads as

6 $$\begin{aligned} \begin{aligned} f^{i+1} =&\bigg (\sum \limits _{m,n} {{\,\mathrm{\mathrm {D}}\,}}b_{f^i} {{\,\mathrm{\mathrm {M}}\,}}{ {{\,\mathrm{\mathrm {D}}\,}}^*b_{f^i} } \bigg )^{-1} \\&\bigg ( \sum \limits _{m,n} {{\,\mathrm{\mathrm {D}}\,}}b_{f^i} {{\,\mathrm{\mathrm {M}}\,}}\left( {{\,\mathrm{\mathrm {A}}\,}}u^{i+1} - b_{f^i} + \langle {{\,\mathrm{\mathrm {D}}\,}}b_{f^i},f^i\rangle \right) \bigg ). \end{aligned} \end{aligned}$$

Note that this is computationally inexpensive, as it requires one only to invert a $2\times 2$ matrix for each projection angle.

#### Reconstruction sub-problem

The second sub-problem emerging from the joint minimization problem solves for *u*. By warping the projection images with the shift estimate $f^i$, Equation (2) simplifies to

7 $$\begin{aligned} \min _{u\in {{\,\mathrm{\mathbb {R}}\,}}^{zmn}} \tfrac{1}{2} \Vert {{\,\mathrm{\mathrm {M}}\,}}( {{\,\mathrm{\mathrm {A}}\,}}u - b_{f^i} )\Vert _2^2+ \lambda \Vert \nabla u\Vert _{2,1}. \end{aligned}$$

To apply an effective optimization procedure, we first transform this minimization problem into a saddle-point problem by dualizing both the data fidelity and regularization term using the Legendre–Fenchel transform. This yields the following sub-problem in *u* and the two dual variables $p \in {{\,\mathrm{\mathbb {R}}\,}}^{3 \times z m n}$ and $q \in {{\,\mathrm{\mathbb {R}}\,}}^{n_{\alpha }m n}$

8 $$\begin{aligned} \begin{aligned} \min _{u\in {{\,\mathrm{\mathbb {R}}\,}}^{zmn}} \max _{\begin{array}{c} p\in {{\,\mathrm{\mathbb {R}}\,}}^{3 \times z m n},\\ q \in {{\,\mathrm{\mathbb {R}}\,}}^{n_{\alpha }m n} \end{array}}\big \{&\langle {{\,\mathrm{\mathrm {M}}\,}}({{\,\mathrm{\mathrm {A}}\,}}u - b_{f^i}), q \rangle - \tfrac{1}{2} \Vert q \Vert _2^2 + \\&\langle \nabla u , p \rangle - \delta _{\Vert \cdot \Vert _{2,\infty } \le \lambda }(p)\big \}, \end{aligned} \end{aligned}$$

where $\delta _{\Vert \cdot \Vert _{2,\infty } \le \lambda }$ denotes the indicator function of the ball $\Vert \cdot \Vert _{2,\infty } \le \lambda$. This saddle-point problem is solved using a primal-dual algorithm (Chambolle and Pock 2011) with diagonal preconditioning (Pock and Chambolle 2011) to reconstruct *u*.

Optimizing solely Equation 8 would not provide a reliable reconstruction *u*; therefore, exactly one primal-dual iteration is performed before recomputing the shift *f*. Solving jointly for both sub-problems in *f* and *u* is summarized in detail in algorithm 1. In each iteration, the mismatched projection images *b* are translated by the current shift $f^i$. This is followed by the primal-dual updates for $u^{i+1}$, $p^{i+1}$ and $q^{i+1}$. The update steps for the dual variables are followed by implicit steps arising from the proximal maps. Note that the definition of a proximal mapping for a function *h* with step size $\eta \in {{\,\mathrm{\mathbb {R}}\,}}^+$ (Beck 2017) reads as

9 $$\begin{aligned} {{\,\mathrm{\mathrm {prox}}\,}}_{\eta h} (x) = \mathop {\mathrm {arg\, min}}\limits _u \tfrac{1}{2} \Vert u - x \Vert ^2 + \eta h(u), \end{aligned}$$

which is a projection operator for the indicator function. The proximal maps for both the data term and the indicator function are shown in more detail in the Appendix. Finally, the shift $f^{i+1}$ is computed from the new reconstruction $u^{i+1}$. These steps are carried out for *N* iterations.

### Datasets

#### Synthetic data

With inverse problems in imaging applications and biological settings, it is often hard to evaluate an algorithm owing to the lack of ground truth data. Therefore, we apply FLARA in a first step to a synthetic 3D phantom to validate the shift computation and whether it is in accordance with the reconstruction. The phantom is inspired by the well-known Shepp–Logan phantom (Rudin et al. 1974) and thus consists of a few basic geometric shapes such as cubes, cuboids, spheres and ellipsoids that are assembled randomly in a volume of size $512\times 512 \times 512$ pixels. To simulate a realistic measurement scenario in electron tomography, the projection data were generated only for a limited tilt angle series covering $\alpha _i \in \{-60^\circ , -59^\circ , \dots , 60^\circ \}$. Artificial shifts $f_i$ were drawn from a standard normal distribution and were then induced in both horizontal and vertical directions on each projection image. Applying algorithm 1 results in a reconstructed volume and a computed two-dimensional shift for each projection image, where an actual ground truth exists for this setting. Further, we want to compare the reconstruction quality and, in particular, the shift computation to a different joint approach. Therefore, the joint iterative reconstruction and re-projection method proposed by Gürsoy et al. (2017) is used, where the authors alternate between shift correction based on cross-correlation and reconstruction in an iterative scheme. A major motivation to use this method to compare our approach against is the fact that it also assumes two underlying shift components in the projection images, whereas other works model the shift within the object. Furthermore, a huge advantage is its availability in the open-source project TomoPy (Gürsoy et al. 2014). Moreover, a comparison with IMOD (Jayasimhan et al. 1996) is also included, which is obtained from using the SIRT algorithm after pre-aligning the projection images using cross-correlation.

#### C57BL/6J and NOD mice

The algorithm was then qualitatively tested on samples containing pancreatic islets of a healthy female C57BL/6J mouse and a female NOD mouse. The healthy mouse was sacrificed at 15 weeks with a blood glucose value of 119 mg/dl, and the NOD mouse was sacrificed at 35 weeks with a blood glucose value of 123 mg/dl. Pancreatic tissue containing endocrine and exocrine pancreas was embedded for further electron microscopical investigations. The endocrine pancreas consists of islets comprising alpha cells, beta cells and delta cells.

#### Electron microscopy

Pancreatic tissue was fixed in 2.5% (wt/vol) glutaraldehyde and 2% (wt/vol) paraformaldehyde in 0.1 M cacodylate buffer, pH 7.4, for 2 h, post-fixed in 1% (wt/vol) osmium tetroxide for 2 h at room temperature (RT). After dehydration (dehydrated in graded series of ethanol), tissues were infiltrated (ethanol and TAAB epoxy resin, pure TAAB epoxy resin) and placed in TAAB epoxy resin (8 h), transferred into embedding moulds, and polymerized (48 h, 60 $^\circ$ C). Ultrathin sections (70 nm and, for electron tomography, 300 nm) were cut with a UC 7 Ultramicrotome (Leica Microsystems, Vienna, Austria) and stained with lead citrate for 5 min and platinum blue (Inaga et al. 2007) for 15 min.

Electron micrographs and electron tomograms of islets were taken using a Tecnai G2 transmission electron microscope (FEI, Eindhoven, Netherlands) with a Gatan ultrascan 1000 charge coupled device (CCD) camera (-20 $^\circ$ C, acquisition software Digital Micrograph, Gatan, Munich, Germany). Acceleration voltage was 200 kV for electron tomography on 300-nm-thick sections. Electron tomograms were generated by the use of the SERIAL EM programme (Mastronarde 2003), and the measured tilt angles covered a span of ±60$^\circ$ with tilt angle increments of 1$^\circ$ resulting in 121 projection images with a magnification of $3,500\times$.

Scanning transmission electron microscopy (STEM) imaging mode of a field emission scanning electron microscope (ZEISS FE-SEM Sigma 500) with an acceleration voltage of 15 kV in combination with ATLAS TM was used to perform imaging on large areas of beta cells with high-resolution AZoNano (2021). This provides an overview of a more extensive area to better localize sections imaged with electron microscopy and tomography within the pancreas. Furthermore, reversing the procedure by first obtaining an overview of the entire section followed by subsequent electron tomography of a selected area of interest will be integrated into our future workflow in the context of correlative electron microscopy.

## Results and discussion

**Fig. 1:**
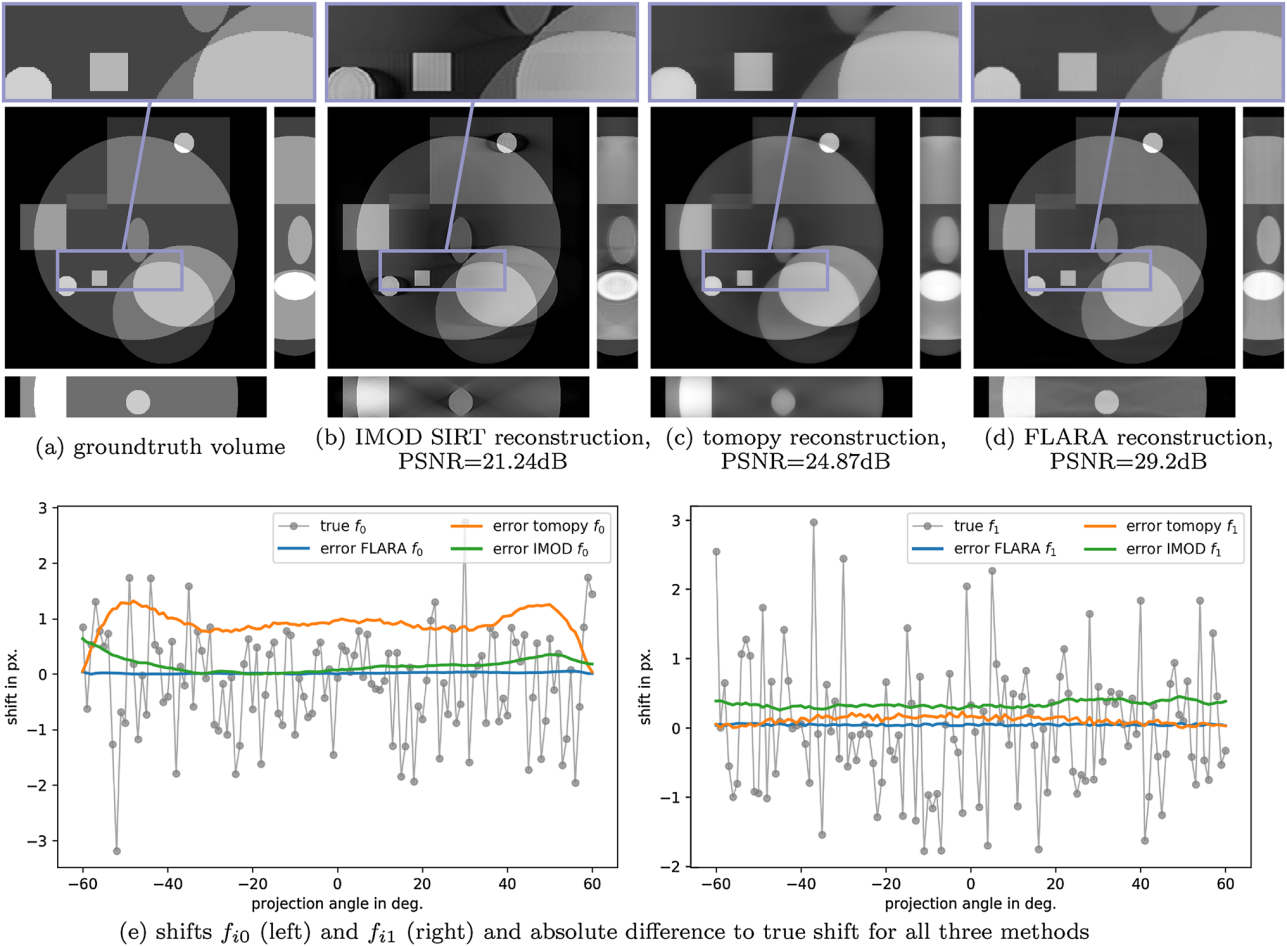
Reconstruction and shift computation for a synthetic phantom (best viewed on screen). The top row shows the ground truth (**a**) and the reconstructions with IMOD’s SIRT algorithm with a PSNR of 21.24 dB (**b**), the toolbox TomoPy with a PSNR of 24.87 dB (**c**) and FLARA with a PSNR of 29.2 dB (**d**). For all reconstructed volumes, central virtual slices of each orthogonal direction are shown. The bottom row shows true shifts for both horizontal ($f_0$) and vertical ($f_1$) shift components, together with the respective absolute differences for all three methods (**e**)

**Table 1:** Comparison of the joint algorithm by Gürsoy et al. (2017) (TomoPy), IMOD’s alignment protocol and FLARA regarding shift computation

	$\text {MSE}$ ± std. for $f_0$	$\text {MSE}$ ± std. for $f_1$	$\text {MAE}$ ± std. for $f_0$	$\text {MAE}$ ± std. for $f_1$	$\text {max. AE}$ for ${f_0}$	$\text {max. AE}$ for ${f_1}$
Tomopy (Gürsoy et al. 2017)	0.8324 ± 0.3576	0.0125 ± 0.012	0.913 ± 0.2228	0.1117 ± 0.0554	1.3182	0.229
IMOD (Kremer et al. 1996)	0.0018 ± 0.0677	0.0372 ± 2.5446	0.16 ± 0.1305	0.3399 ± 0.0449	0.6388	0.45
FLARA	0.0003 ± 0.0006	0.00198 ± 0.00099	0.0188 ± 0.0126	0.0445 ± 0.011	0.055	0.062

Mean squared error (MSE) and standard deviation (std.) as well as mean absolute error (MAE) and maximum absolute error (max. AE) for both shift components with respect to the true shift are denoted for all methods. Units for absolute errors are in pixels

Before presenting reconstruction results obtained for pancreatic healthy C57BL/6J and NOD samples, we want to test our algorithm on a synthetic dataset. This allows us to validate the shift computation against known induced shifts in projections of a generated phantom, which is not possible in real-world applications.

### Evaluation on synthetic data

The resulting reconstruction and shift computation for the synthetically generated phantom is depicted in Fig. 1. The top row shows the ground truth (a), the reconstructions obtained from IMOD’s SIRT algorithm (b), the joint iterative alignment method based on cross-correlation by Gürsoy et al. (2017) (c) and FLARA (d). The peak signal-to-noise ratio (PSNR) values of the reconstructed volumes are 21.24 dB, 24.87 dB and 29.2 dB, respectively – note that using clean projection data in the same reconstruction procedure without shift computation only marginally increases the PSNR to 29.41 dB. We notice a clearly improved reconstruction quality with respect to both the SIRT reconstruction and the joint reconstruction (Gürsoy et al. 2017) in terms of PSNR. This is confirmed by the enhanced visual quality exhibited in sharper edges and contours. This difference highlights the importance of carefully selecting a correction scheme for shifted projection images. Shift correction based on the linearization scheme delivers improved results compared with a cross-correlation scheme, which can be explained by the fact that our method obtains global information from all projection images. The bottom row shows for both shift components the true shift for each projection angle $\alpha _i$, accompanied by the absolute error of the computed with respect to the true shift for each projection angle for all three methods, recalling that shifts are computed with IMOD’s alignment protocol, the joint algorithm from Gürsoy et al. (2017) (termed ‘TomoPy’ in this work for convenience only), and with FLARA. The mean squared error (MSE), mean absolute error (MAE) and maximum occurring absolute error (max. AE) for both shift components are denoted in Table 1. The quantitative values remarkably imply that the remaining error from our proposed algorithm FLARA is at a sub-pixel level, even though it cannot be eliminated completely, while also showing that FLARA delivers better quantitative results. The mean and maximum absolute error in the shift components from IMOD and the TomoPy algorithm is also at a sub-pixel level for component $f_1$; however, for $f_0$ a substantial deviation from the true shift can be observed. In general, remaining shift errors are due to the fact that the shift computation depends on the current reconstruction, which naturally deviates slightly from the ground truth phantom owing to the limited angle setting. Further, due to the fact that the alignment procedure cannot be used to recover the absolute ground truth position of the phantom in 3D, the difference in the horizontal component $f_0$ of the computed shift depends on the tilt angle, whereas the vertical component $f_1$ will contain only a minor constant offset. This effect has also been described in literature (Dengler 1989; Gürsoy et al. 2017).

The reconstruction obtained with FLARA still contains some artefacts, which is a well-known issue in limited-angle tomography. In this scenario, only 121 projections covering angles of ±60$^\circ$ are acquired instead of 181 projections covering all angles of ±90$^\circ$, which would be required to generate an artefact-free reconstruction. However, the quality of the reconstruction is reasonably good, as indicated by the reported PSNR value, which is in accordance with other well-established iterative reconstruction algorithms (Goris et al. 2013; Chen et al. 2013). Improving the limited angle problem is a different research question in itself, and there are numerous works that tackle this (Bubba et al. 2019; Würfl et al. 2018; Gu and Ye 2017). However, the purpose of this work is to demonstrate the shift computation in a joint manner with the reconstruction. The method of computing the shift can naturally be included in any other iterative reconstruction framework.

### Application to C57BL/6J and NOD mice

**Fig. 2:**
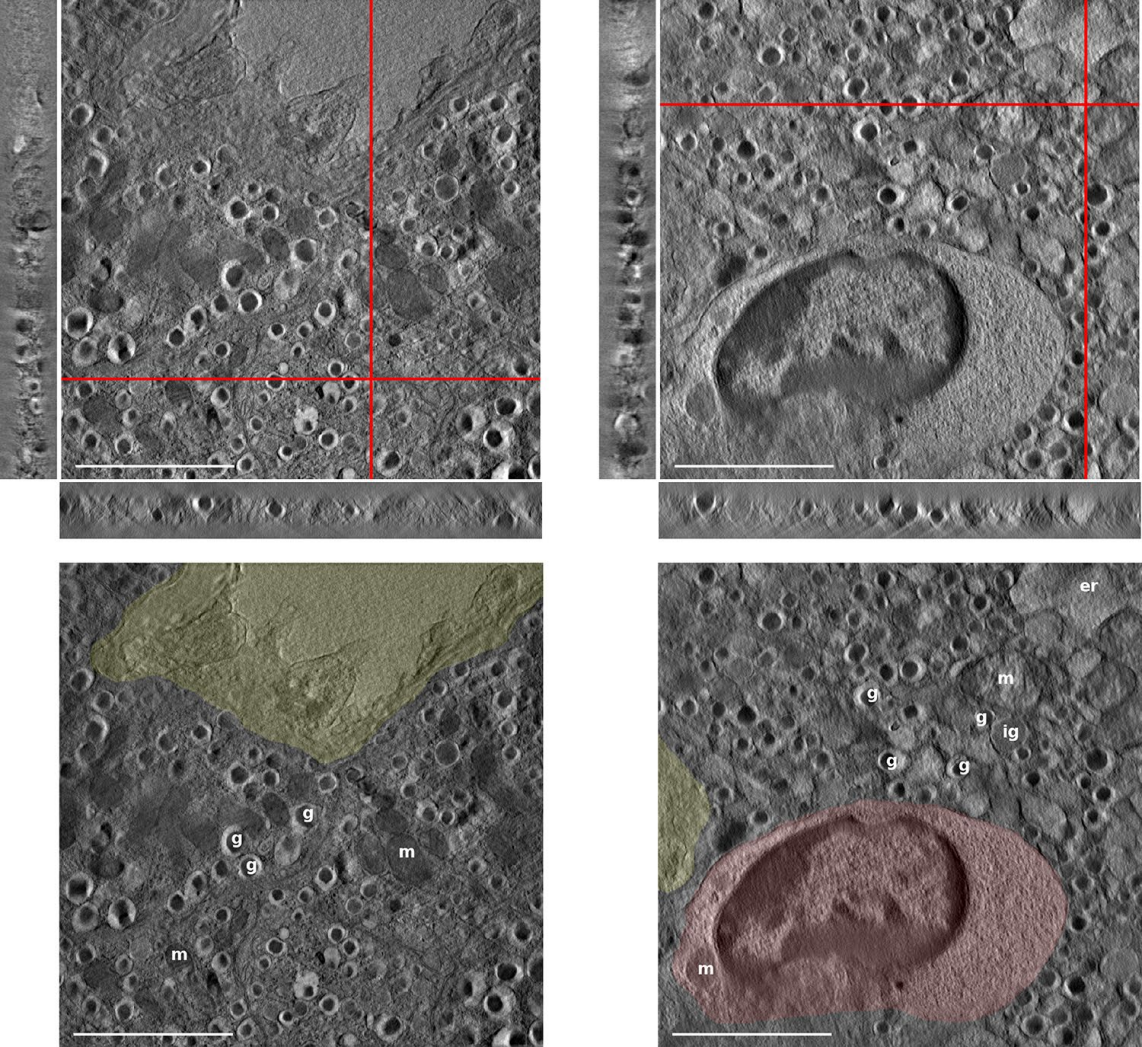
Virtual slices of tomographic reconstructions of 300-nm-thick sections of healthy C57BL/6J (top left) and pre-diabetic NOD (top right) pancreata. For both reconstructions, virtual slices through each orthogonal direction are shown, as indicated by the in-plane red lines. The presence of an immune cell that attacks the beta cells can be clearly seen within the NOD islet, which leads to inhibited insulin production. The bottom row shows the central virtual slice with overlaid components of interest, where the presence of a vessel is highlighted in yellow, and the red colour denotes an invading immune cell. Lowercase letters ‘g’, ‘ig’, ‘m’ and ‘er’ are labels for granules, immature granules, mitochondria and endoplasmic reticulum, all of which are organelles found within the cytoplasm. The insulin-secretory granules are particularly interesting in this analysis as they are the insulin-containing units of the beta cells, while immature granules contain proinsulin, a precursor of insulin. Scale bar is 2$~\upmu$m

**Fig. 3:**
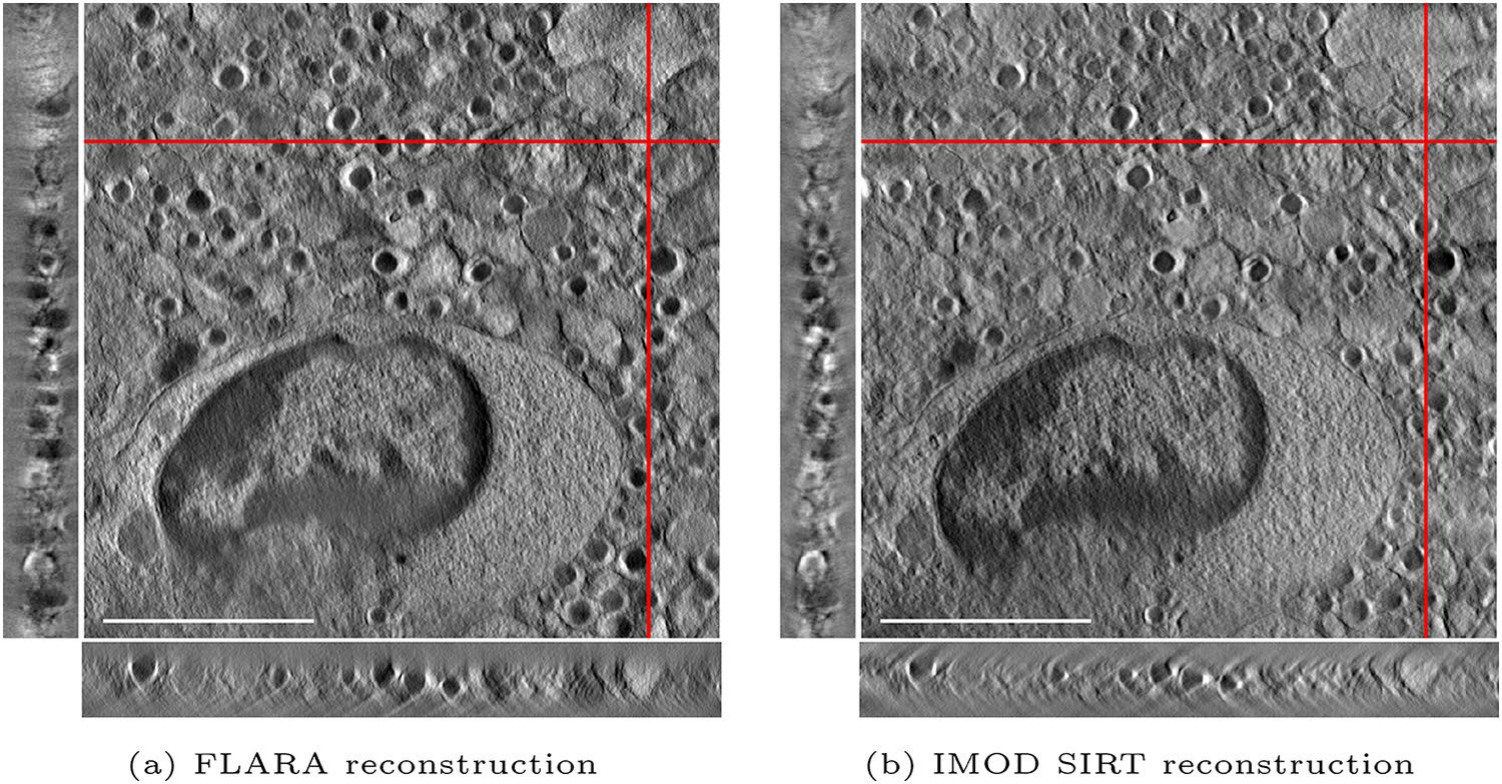
Virtual slices of 300 nm reconstructions of pre-diabetic NOD pancreata using FLARA (**a**) and IMOD’s SIRT reconstruction following fiducial-less alignment based on cross-correlation prior to reconstruction (**b**). For both reconstructions, the same virtual slices through each orthogonal direction are shown, as indicated by the in-plane red lines. Scale bar is 2$~\upmu$m

The algorithm was then applied to real-world electron tomography data to obtain qualitative results on acquired tilt series. Virtual sections of tomographic reconstructions of 300-nm-thick sections from healthy C57BL/6J and NOD mice are shown in Fig. 2, left and right, respectively. For both reconstructions, virtual slices through each of the three orthogonal directions are shown, where selected visualized virtual slices are indicated by the red lines in plane. The choice of depicted virtual slices is arbitrary, ensuring only that the profile of beta cell granules is visible to indicate the quality of the reconstruction throughout the entire volume. Moreover, a qualitative comparison of the reconstructed pancreatic NOD sample using IMOD’s SIRT algorithm following fiducial-less alignment prior to reconstruction and FLARA is shown in Fig. 3. In both reconstructions, all ultrastructural details, especially the immune cells’ interaction with the insulin-secretory granules in the beta cell, can be clearly seen. However, it is noteworthy that the IMOD reconstruction contains some artefact-prone structures visible in the orthogonal view that are most likely originating from the alignment procedure.

In the second row of Fig. 2, the central virtual slice of each reconstruction is visualized with an emphasis on important key components of beta cells such as blood vessels (coloured in yellow), insulin-secretory granules (g), immature granules (ig), mitochondria (m) and endoplasmic reticulum (er). The reconstructed sample of the healthy C57BL/6J mouse contains insulin-secretory granules and also depicts a blood vessel (coloured in yellow) but does not contain any immune cells. This reflects the general state of healthy C57BL/6J beta cells.

As highlighted in red in Fig. 2, the beta cell of the NOD mouse is invaded by an immune cell that reaches the beta cells via blood vessels (Zinselmeyer et al. 2018). This is a good indicator for the active process in the development of type 1 diabetes, where cytotoxic immune cells attack beta cells in the pancreas. This leads to beta cell stress followed by apoptosis and inhibition of insulin secretion (Jayasimhan et al. 2014). Even though the direct causes of this autoimmune attack are still unknown, the phenotypical characterization obtained by electron tomography can be used to gain more insight into this process. It is worth mentioning that the NOD mouse is pre-diabetic and thus has normoglycemic blood glucose. This is due to the fact that it is generally difficult to find remaining islets within diabetic mice; for this reason, we resorted to investigating islets of a pre-diabetic mouse, which can already exhibit the active stage of type 1 diabetes development.

**Fig. 4:**
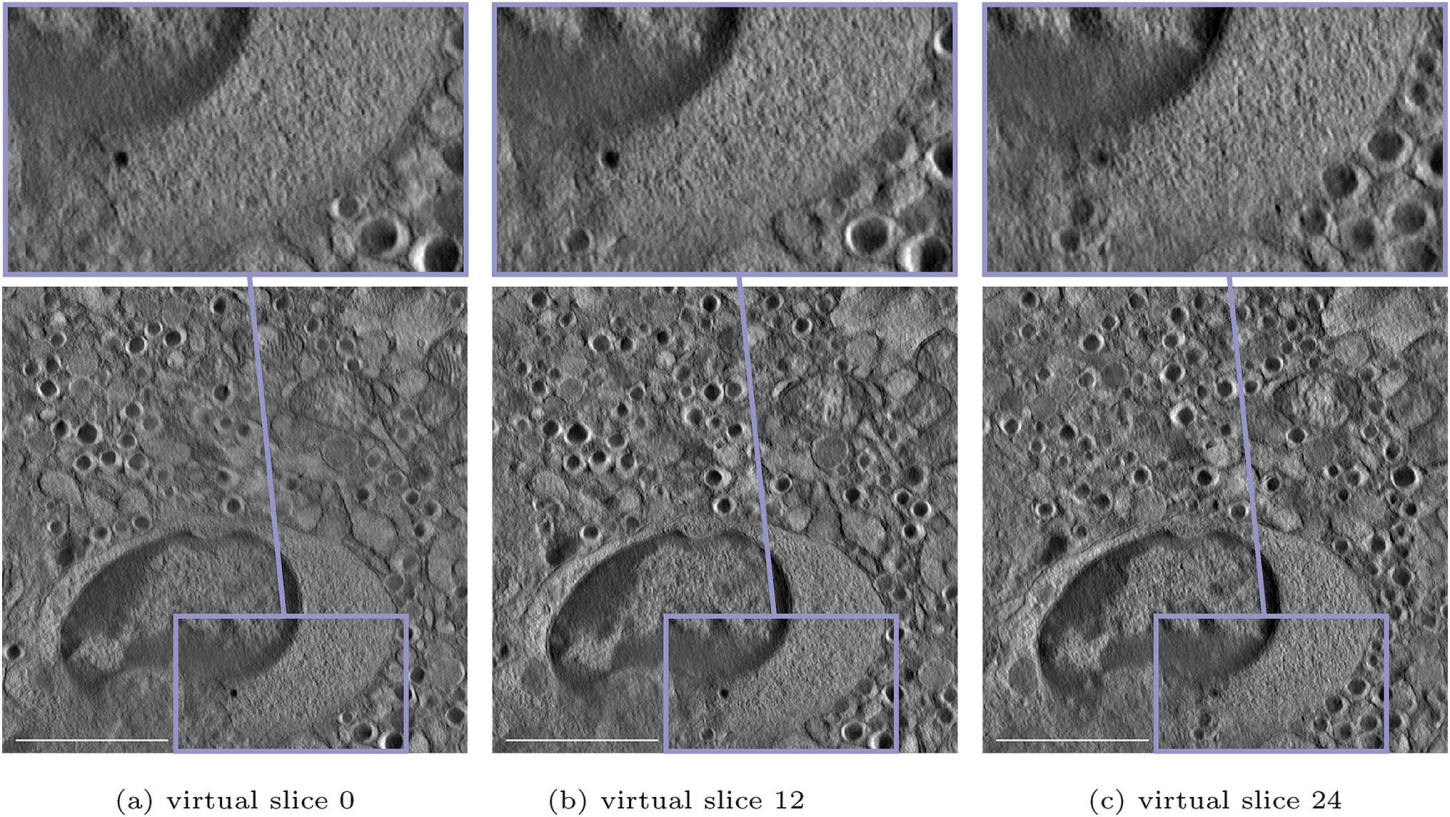
Depiction of three distinct virtual slices of the reconstructed volume of the NOD beta cell with an enlarged view of the selected area of interest for each slice. For the lower two granules shown in the zoomed area, we assume some form of contact or interaction with the immune system, but the granules have probably not been ingested. However, the black circular structure above is strongly suspected to be an enclosed granule internalized within the cytoplasm of the immune cell, as hinted by the enlarged view, especially in virtual slice 12. Scale bar is 2$~\upmu$m

**Fig. 5:**
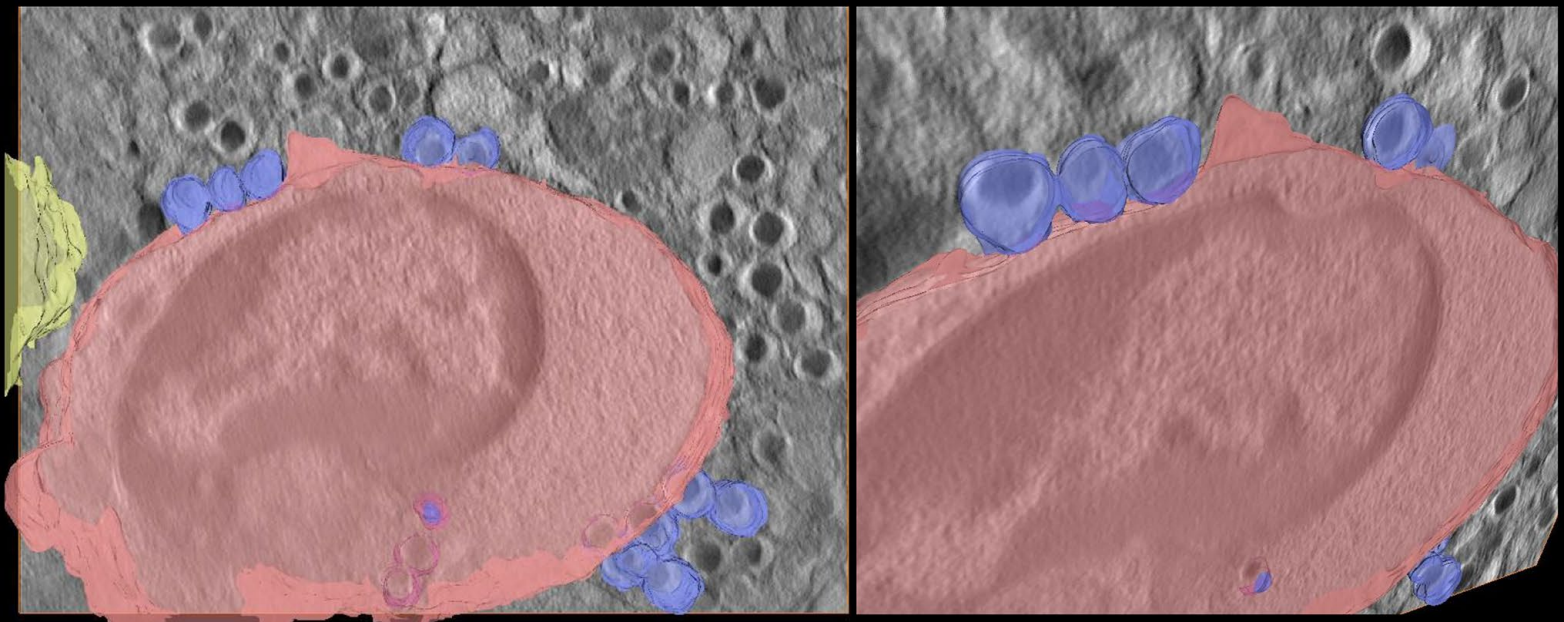
Different views on a 3D visualization of the internalization of insulin-secretory granules (blue) in the cytotoxic immune cell (red) showing the ongoing and already occurred uptake of the granules into the cytoplasm. The presence of the vessel near the immune cell within the beta cell is highlighted in yellow

Interestingly, an internalization of an insulin-secretory granule into the cytoplasm of the immune cell and a further interaction with two granules can be observed visually when looking at different virtual slices of the reconstructed volume of the NOD mouse in Fig. 4. An enlarged view of the area of interest is also included. Although the exact boundaries of the immune cell, especially in the lower part of the reconstruction, cannot be fully delineated, it is highly probable to assume that the process of internalization has been captured. The invading immune cell certainly has ingested the upper granule, and there seems to be a close interaction with the two granules beneath. This is presumably a key step in perpetuating the autoimmune attack on the beta cell and will continue to maintain the ongoing autoimmune process. Note that reconstructing 300-nm-thick samples allows for more information along the third dimension, which makes such a visual analysis of the interaction between immune cell and beta cell more informative. The three virtual slices are chosen from the entire volume such that they are not adjacent to contain more information of the ingestion and interaction of the immune cell with the insulin-secretory granules. An additional visualization of the described process is shown in Fig. 5, where the internalization processes of the insulin-secretory granules into the cytotoxic immune cell that already occurred and that are currently ongoing are highlighted in different colours. Owing to damage inflicted on the NOD sample while inserting it into the microscope grid, it was not possible to fully exploit the benefit of the STEM technology in this case. Therefore, we resorted to investigating an adjacent section with STEM. Nevertheless, this allows for a more profound analysis by locating the beta cell and the immune cell within the islet, whereby more information can be found in the supplementary material.

### Discussion and outlook

In this paper, we present FLARA, a fiducial-less alignment and reconstruction algorithm for electron tomography, and in parallel present a phenotypical characterization of the pathophysiologic processes of type 1 diabetes, a life-threatening disease. To the best of our knowledge, this is the first time that the internalization of beta cell secretory granules in the immune cell’s cytoplasm has been directly visualized, a process that will most likely ignite further autoimmune processes.

FLARA offers some major advantages over standard reconstruction following alignment based on fiducial markers. First and foremost, it enables fiducial-less alignment and thus alleviates the need to label the sample with fiducial markers in a pre-processing step. Even though an alignment prior to reconstruction based on fiducial markers delivers precise reconstruction results and is considered to be the gold standard, we argue that it is always desirable to omit a pre-processing step if comparable results can still be achieved, because it saves time and material resources. Moreover, the computed shift is expected to get more accurate in subsequent iterations as the reconstruction improves, which in turn leads to an enhanced alignment accuracy. For the conventional procedure of first aligning projection images followed by reconstruction, the possibility of further refinement or corrections to the initial alignment does not exist once the reconstruction has started. Additionally, it is worth mentioning that our method of computing the shift is a global alignment technique, as each shift component is influenced by all projection images and their spatial finite differences. This is also beneficial compared with other fiducial-less reconstruction techniques based on cross-correlation where successive images are aligned only to each other and possible alignment errors can accumulate. Finally, it is noteworthy that the shift is not estimated in an iterative update scheme, but is re-computed in each iteration based on the current reconstruction and the linearized warping operator. Successive iterations thus involve a re-computation of the shift based on the updated reconstructed volume instead of a gradient-based update. The proposed algorithm FLARA can naturally be used for any reconstruction task that involves joint alignment and reconstruction of projection images. In this work, it was used to visualize the interaction between immune cells and beta cells in the pancreas in NOD mice; however, it is also equivalently well suited to investigate any other cell-to-cell or cell-to-tissue interaction. To highlight the versatility and generality of our algorithm, there are numerous further possible applications such as visualizing the interaction between macrophages and endothelial cells or the plaque formation in arteriosclerosis, to mention but a few.

The browser link for the STEM atlas of an adjacent slice of the demonstrated NOD sample and the accompanying source code is available online^1^. The implementation of FLARA embeds ASTRA’s projection operators (Aarle et al. 2015) into a PyTorch (Paszke et al. 2019) framework, and it provides modularity and flexibility compared with existing reconstruction programmes. The source code can be variably extended to contain further quantification and direct post-processing routines that may tackle other research questions such as machine-learning tasks on the granules in the beta cells.

As a future direction, we plan to use FLARA to analyse the interaction between the immune cells and beta cells within the pancreas in NOD mice in more detail. It is of particular interest to investigate whether there are differences in this destructive interaction amongst different stages of type 1 diabetes, where the pathology and the mechanisms that trigger autoimmune attacks are not yet understood in detail. Combined with the established STEM workflow, we plan to set up a routine allowing for a detailed analysis of the temporal evolution of pancreatic islets in NOD mice.

### Supplementary Information

Below is the link to the electronic supplementary material.

Supplementary file 1 (pdf 2729 KB)

## Appendix

### Proximal maps for the dualized data term and regularizer

To derive a closed-form expression of the proximal operators for the dualized data term and regularizer, we first recall the definition of proximal map for a function *h* which reads as

$$\begin{aligned} {{\,\mathrm{\mathrm {prox}}\,}}_{\eta h} (x) = \mathop {\mathrm {arg\, min}}\limits _u \tfrac{1}{2} \Vert u - x \Vert ^2 + \eta h(u), \end{aligned}$$

where $\eta \in {{\,\mathrm{\mathbb {R}}\,}}^+$ denotes the step size. For the data term, the function *h* is the squared $\ell _2$ norm of the dualized least squares data fidelity norm. The proximal map for $h(q)=\tfrac{1}{2}\Vert q \Vert _2^2$ is thus computed by

$$\begin{aligned} {{\,\mathrm{\mathrm {prox}}\,}}_{\frac{\sigma _2}{2} \Vert q \Vert _2^2} (q) = \frac{q}{1 + \sigma _2}. \end{aligned}$$

For the dualized regularizing function, the proximal map is computed for the indicator function and reads as

$$\begin{aligned} {{\,\mathrm{\mathrm {proj}}\,}}_{\Vert \cdot \Vert _{2,\infty } \le \lambda } (p) = \frac{p}{\max \big (1, \frac{|p |}{\lambda }\big )}, \end{aligned}$$

where |*p*| denotes the pixel-wise $\ell _2$ norm over the three finite difference components.

## Footnotes

1 Source code and browser link for STEM atlas: https://github.com/leabogensperger/tem-reco
