# Supplementary material for: A joint alignment and reconstruction algorithm for electron tomography to visualize in-depth cell-to-cell interactions
Source: Histochem Cell Biol. 2022 Mar 23;157(6):685–96. doi: 10.1007/s00418-022-02095-z (PMC9124659; doi:10.1007/s00418-022-02095-z)
Supplement: Supplementary file 1 — (pdf 2729 KB) [file 418_2022_2095_MOESM1_ESM.pdf]

Supplementary Material to the Manuscript

"A Joint Alignment and Reconstruction Algorithm for Electron Tomography to Visualize In-Depth Cell-to-Cell Interactions"

Lea Bogensperger* Erich Kobler† Dominique Pernitsch‡ Petra Kotzbeck§
Thomas R. Pieber¶ Thomas Pock* Dagmar Kolb‖‡

Corresponding authors:

Dagmar Kolb
dagmar.kolb@medunigraz.at

Thomas Pock
pock@icg.tugraz.at.

1 IMOD Reconstruction of Synthetic Dataset

Figure 1 shows results for the comparison between FLARA and IMOD [3] in terms of reconstruction quality and shift computation. The reconstruction in IMOD was done using the simultaneous iterative reconstruction technique (SIRT) based on aligned projection data using cross-correlation, where the estimated shifts were extracted from a log file provided by the program. Comparing both shift components and their accompanying absolute error for FLARA and IMOD it can clearly be seen that a systematic error is present in the shift computation for the first component f_0 with IMOD, which can also be observed for the tomopy reconstruction in the manuscript. On the other hand, the second component f_1 exhibits an offset for all angles in the absolute error, which has also been observed with the tomopy [2] algorithm. This can be attributed to the fact that it is not possible to obtain the exact position of the imaged object, and a the resulting error will be visible as a constant offset and an additional systematic error related to geometric circumstances, depending on how the object is tilted.

The reconstruction quality in terms of PSNR and visual inspection for IMOD is not competitive when compared to FLARA. This is primarily due to the additional regularizer used with FLARA, which enforces piecewise constant regions.

2 STEM Overview

Figure 2 shows a schematic of a STEM acquisition of an adjacent slice to the NOD sample, which provides a good overview on a larger area of the islet embedded in the exocrine pancreas. This imaging modality enables continuous zooming into the islet and guarantees us that the selected section imaged with electron tomography contains areas of interest with regards to cellular interactions within the beta cells, which are shown in the second column of Figure 2.

*Institute of Computer Graphics and Vision, Graz University of Technology, Austria.
†Institute of Computer Graphics, University of Linz, Austria
‡Core Facility Ultrastructure Analysis, Graz, Austria.
§COREMED, Cooperative Centre for Regenerative Medicine, Joanneum Research, Graz, Austria; Research Unit for Tissue Regeneration, Repair and Reconstruction, Medical University of Graz, Austria.
¶Division of Endocrinology and Diabetology, Medical University of Graz, Austria; Center for Biomarker Research in Medicine GmbH, Austria.
‖Gottfried Schatz Research Center for Cell Signaling, Metabolism and Aging, Division of Cell Biology, Histology and Embryology, Medical University of Graz, Austria.

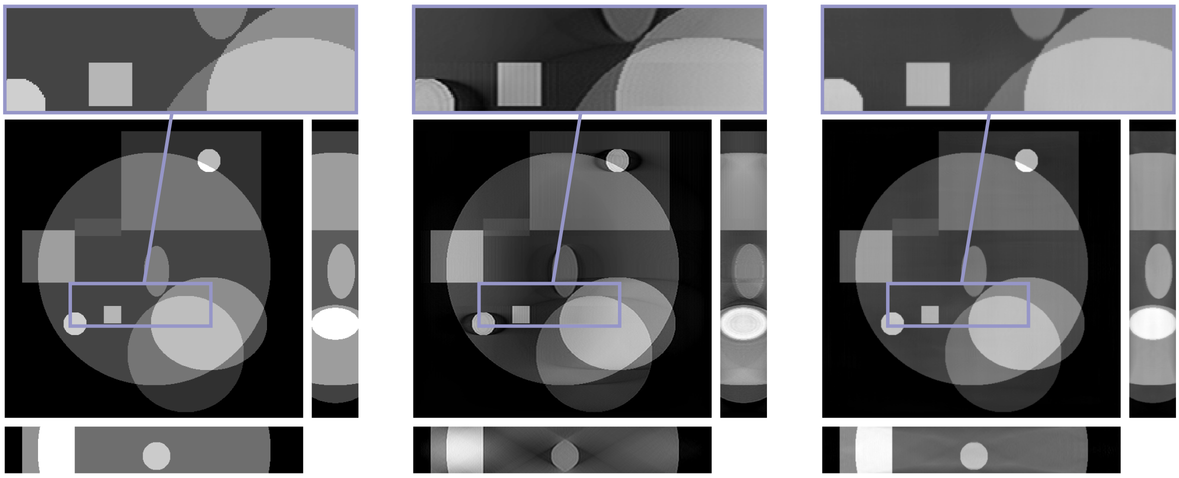

(a) groundtruth volume

(b) IMOD SIRT reconstruction, PSNR=21.24dB

(c) FLARA reconstruction, PSNR=29.2dB

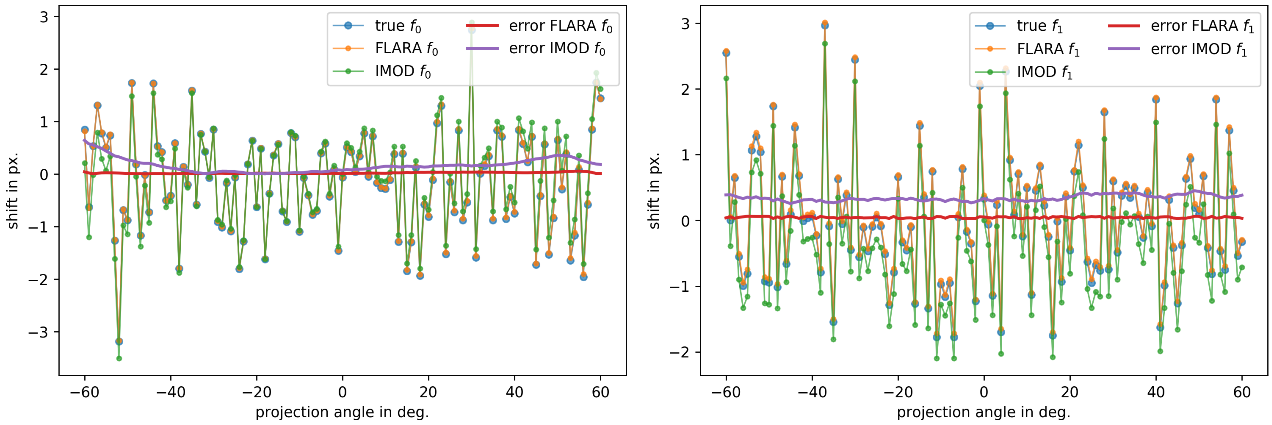

(d) computed shifts f_{i0} (left) and f_{i1} (right) for both methods with absolute difference to true shift

Fig. 1. Reconstruction and shift computation for a synthetic phantom. The top row shows the groundtruth (a), a SIRT reconstruction with IMOD with a PSNR of 21.24 dB (b), and the FLARA reconstruction with a PSNR of 29.2 dB (c). The bottom row shows true and computed shifts for both horizontal (f_0) and vertical (f_1) shift components from FLARA and IMOD together with their respective absolute difference (d)

3 Systematic Error in the Shift Computation

In Figure 3 results from the joint alignment and reconstruction algorithm provided by the tomopy toolbox [1] for rotated input data are depicted.

As presented in the manuscript and in the supplementary in Figure 1, there is a systematic error in the first shift component f_0 (horizontal when looking at the plane of the central reconstruction slice) due to the tilting. A small error in the shift estimation might geometrically accumulate for an alignment procedure based on cross-correlation due to tilting.

References

[1] Doga Gürsoy, Francesco De Carlo, Xianghui Xiao, and Chris Jacobsen. Tomopy: a framework for the analysis of synchrotron tomographic data. *Journal of synchrotron radiation*, 21(5):1188–1193, 2014.

[2] Doga Gürsoy, Young Hong, Kuan He, Karl Hujsak, Seunghwan Yoo, Si Chen, Yue Li, Mingyuan Ge, Lisa Miller, Yong Chu, Vincent De Andrade, Kai He, Oliver Cossairt, Aggelos Katsaggelos, and Chris Jacobsen. Rapid alignment of nanotomography data using joint iterative reconstruction and reprojection. *Scientific Reports*, 7, 09 2017.

[3] James R. Kremer, David N. Mastronarde, and J.Richard McIntosh. Computer visualization of three-dimensional image data using imod. *Journal of Structural Biology*, 116(1):71–76, 1996.

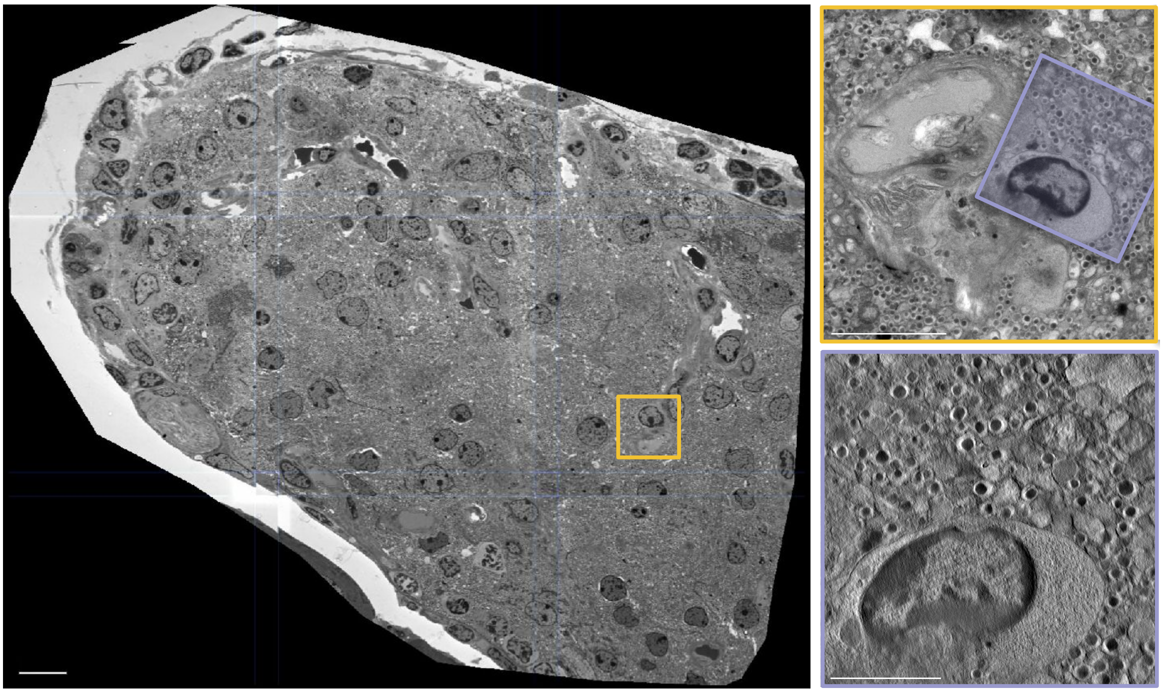

Fig. 2. Overview of a pancreatic islet visualized with STEM (left) providing an overview on a larger area of the islet within an adjacent slice. This enables us to locate our selected area (yellow rectangle) used for electron tomography within the STEM overview, indicating the presence of a vessel nearby the immune cell. The right column on top shows a TEM micrograph of a larger area of the beta cell and the selected area (blue rectangle) used for the tilt series that allows for a more detailed phenotypical characterization of the interaction between the immune cell and the beta cell. On the bottom of the right column, the central virtual slice of the NOD reconstruction is shown. Scale bar is 10 μm (left) and 2 μm (right top and bottom)

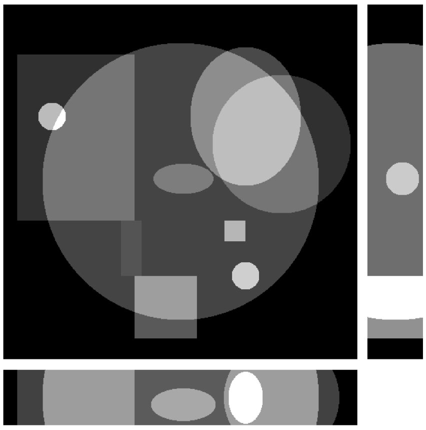

(a) rotated groundtruth volume

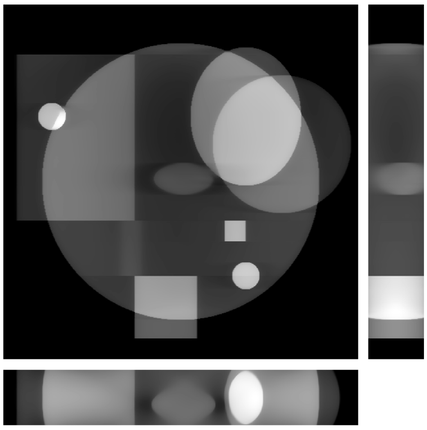

(b) tomopy reconstruction, PSNR=19.08dB

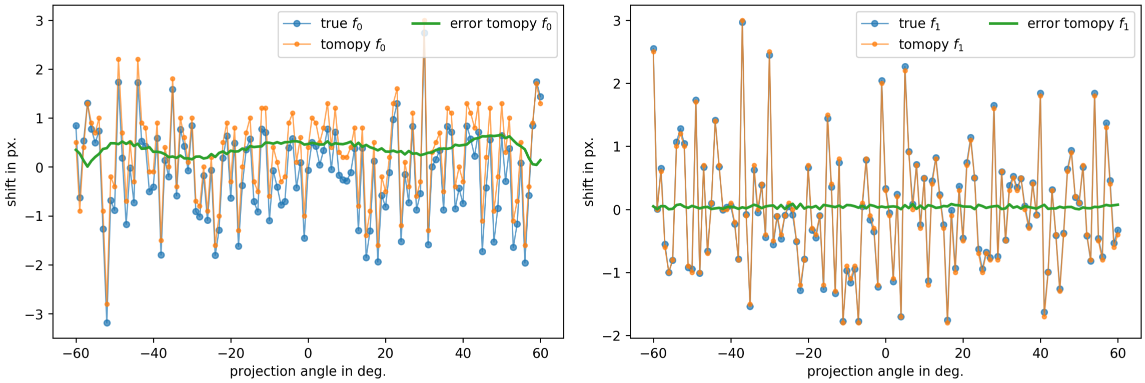

(c) computed shifts f_{i0} (left) and f_{i1} (right) with absolute difference to true shift

Fig. 3. Reconstruction (top) and shift computation (bottom) for rotated data using tomopy
